# Supplementary figures and images for: Aging Model for Analyzing Drug-Induced Proarrhythmia Risks Using Cardiomyocytes Differentiated from Progeria-Patient-Derived Induced Pluripotent Stem Cells
Source: Int J Mol Sci. 2023 Jul 26;24(15):11959. doi: 10.3390/ijms241511959 (PMC10418415; doi:10.3390/ijms241511959)

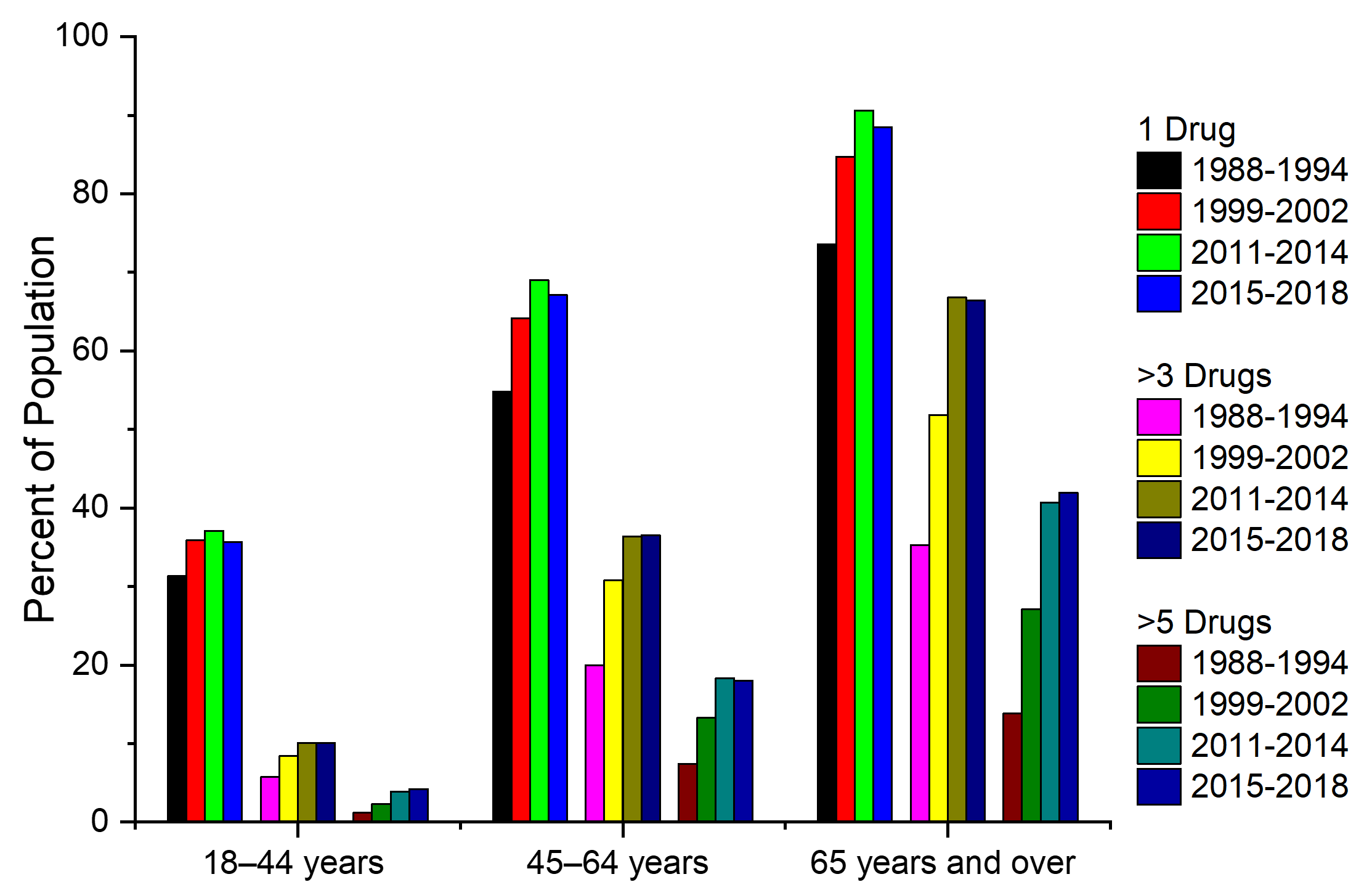

Supplement: Supplementary file 1 [file ijms-24-11959-s001.zip › FigureS1.tif]

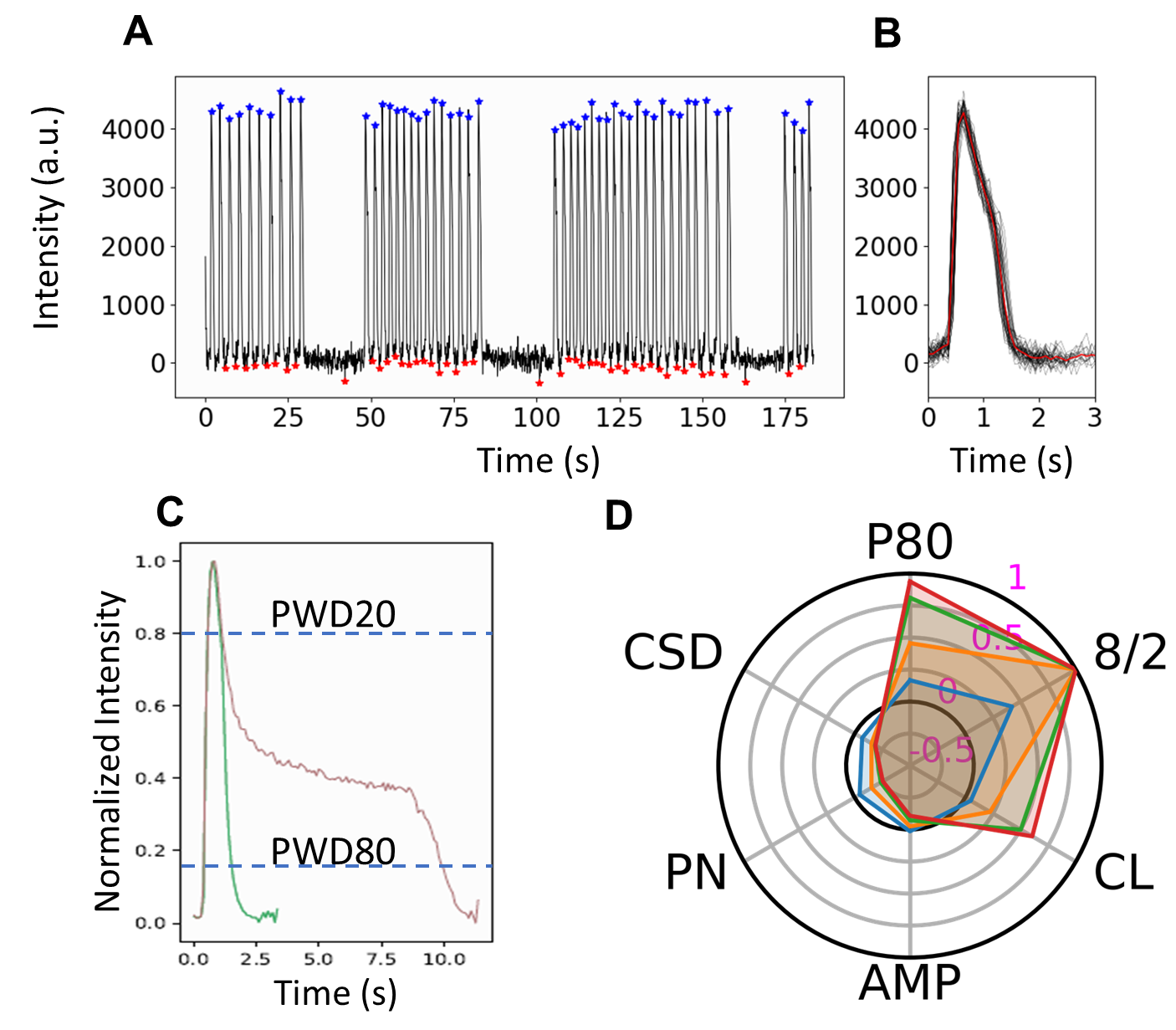

Supplement: Supplementary file 1 [file ijms-24-11959-s001.zip › FigureS2.tif]

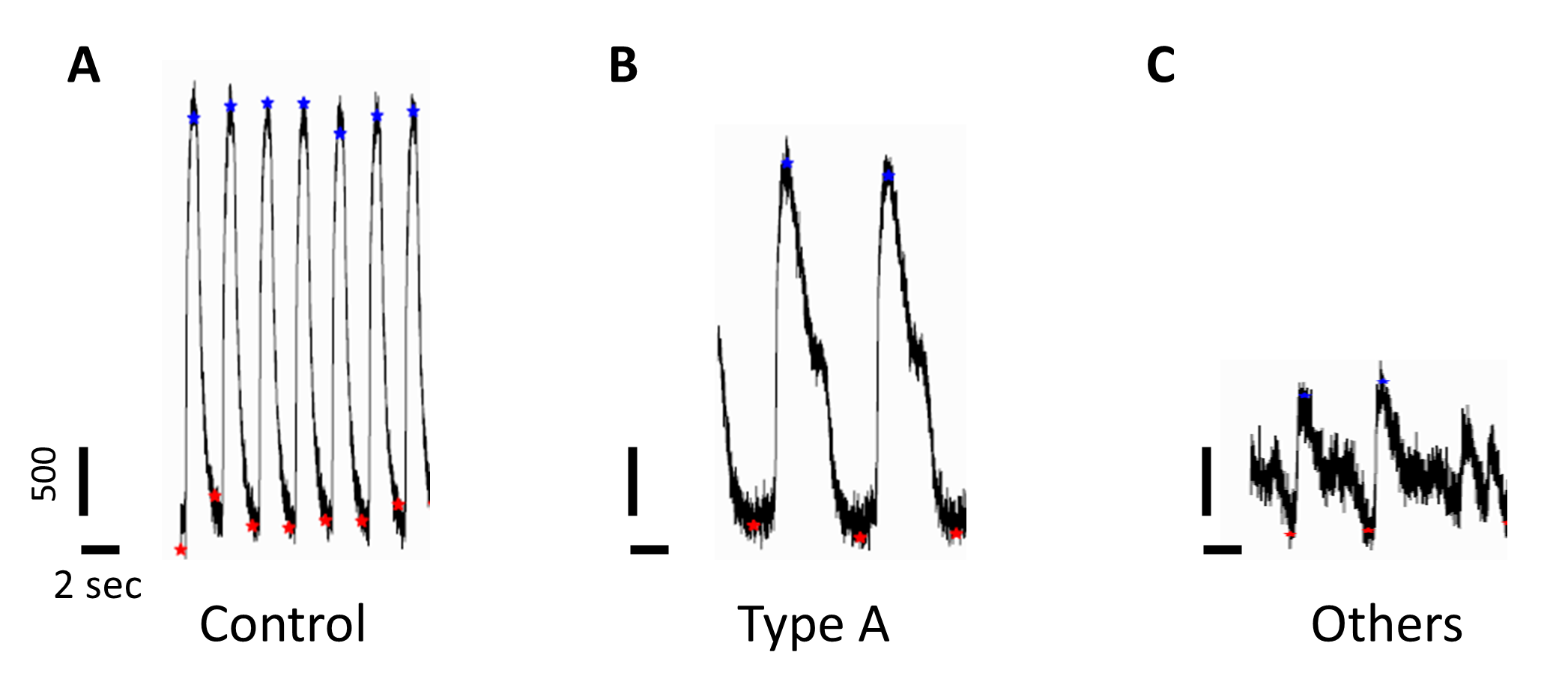

Supplement: Supplementary file 1 [file ijms-24-11959-s001.zip › FigureS3.tif]
